# Automatic extraction of gene-disease associations from literature using joint ensemble learning

**DOI:** 10.1371/journal.pone.0200699

**Published:** 2018-07-26

**Authors:** Balu Bhasuran, Jeyakumar Natarajan

**Affiliations:** 1 DRDO-BU Center for Life Sciences, Bharathiar University Campus, Coimbatore, Tamilnadu, India; 2 Data mining and Text mining Laboratory, Department of Bioinformatics, Bharathiar University, Coimbatore, Tamilnadu, India; Instituto Nacional de Medicina Genomica, MEXICO

## Abstract

A wealth of knowledge concerning relations between genes and its associated diseases is present in biomedical literature. Mining these biological associations from literature can provide immense support to research ranging from drug-targetable pathways to biomarker discovery. However, time and cost of manual curation heavily slows it down. In this current scenario one of the crucial technologies is biomedical text mining, and relation extraction shows the promising result to explore the research of genes associated with diseases. By developing automatic extraction of gene-disease associations from the literature using joint ensemble learning we addressed this problem from a text mining perspective. In the proposed work, we employ a supervised machine learning approach in which a rich feature set covering conceptual, syntax and semantic properties jointly learned with word embedding are trained using ensemble support vector machine for extracting gene-disease relations from four gold standard corpora. Upon evaluating the machine learning approach shows promised results of 85.34%, 83.93%,87.39% and 85.57% of F-measure on EUADR, GAD, CoMAGC and PolySearch corpora respectively. We strongly believe that the presented novel approach combining rich syntax and semantic feature set with domain-specific word embedding through ensemble support vector machines evaluated on four gold standard corpora can act as a new baseline for future works in gene-disease relation extraction from literature.

## Introduction

Advancements in science and technology act as a major influence on the fast increase of scientific publications, especially in the field of biomedicine [1]. Scientific advancements in the research of diseases made potential discoveries in molecular and cellular components and revealed new insights into genetic alterations and signaling pathways [2]. By combining precision medicine, diagnostics and translational research there is an increasing effort and breakthroughs in pinpointing more susceptible biomarkers or improve the efficiency of certain treatments [3]. All these research findings are in a large amount of biomedical literature, in order to keep up with new findings and to generate valid insights researchers need to go through a very difficult, tedious manual reads and analysis. As a systematic solution, biomedical text mining is evolved and generated exceptional results and knowledge discovery in the past years using its ability to process biomedical and scientific literature automatically in large-scale [4].

Some of the well-known applications such as named entity recognition (NER) [5], relation extraction (protein-protein interaction, chemical-disease association) [6,7], identification of bio-events [8] and pathways [9], hypothesis generation [10] made biomedical text mining a crucial part of scientific research. From among this, one of the long-standing goals of computational biology is evidently discovering the roles of candidate genes associated with a specific disease [11]. Researchers approached the problem of this relation extraction task by implementing certain techniques that can be broadly classified as a pattern or rule-based [12], co-occurrence statistics based [13,14] and supervised learning approaches [15–17]. Among these supervised learning approaches are popular and in supervised learning, a set of features that can reflect the relationship between the entities along with a kernel function is used for relation extraction [15–17]. Recently, the studies of relation extraction have been introduced the hybrid approach in which two or more of the above-mentioned approaches are combined to achieve better performance systems [18]. By applying effective relation extraction methodologies to extract the gene-disease associations from it can empower discovery and advancement of patient segment biomarkers and new curative targets [19].

To assist researchers with the vast amount of gene disease associations a large number of curated databases created from the literature using text mining are available. UniProtKB [20], DisGeNET [21], STRING [22], OMIM [23], PharmGKB [24] and CTD [25] are some of the gene-disease association repositories, which employed text mining based procedures for the curation of such associations from biomedical literature.

Potential growth and strong demands of disease associated researchers over the years showed increasing attempts to extract gene-disease relations from biomedical text. Researchers of biomedical text mining approached the problem of gene-disease relation extraction as large-scale mining or supervised machine learning or combining these into a single methodology. Early works of gene-disease relation extraction lacked gold standard corpora, so they reported the results based on existing databases like PharmGKB [24] and CTD [25]. To support the development of such methodologies and to boost the studies on gene-disease associations several text mining approaches have been proposed in the form of methodologies, tools and curated databases. Some of the notable text mining tools that have been released with a core focus on extracting gene disease associations from text have been discussed in the following section.

### Related works

To address the problem of gene-disease relation extraction Bravo et al., proposed a supervised approach BeFree, using text and large-scale data[26]. They used the morpho-syntactic features of text along with dependency kernel and reported real case scenarios and discussed its application in translational research [26]. Pletscher-Frankild, Sune, et al., introduced a dictionary-based tagger combined with co-occurrence scoring and released as DISEASES resource, by integrating text mining along with genome-wide association studies and cancer mutation data [27]. Song et al., developed a comprehensive text mining system PKDE4J, by using Stanford CoreNLP based named entity recognition and rule-based relation extraction [28]. Liu, Rey-Long, and Chia-Chun Shih used degrees of conclusive, rich and focused references to rank gene disease associations using the technique CRFref [29]. Liu et al., focused on the dictionary-based extraction of simple association discovery of multiple concepts like gene, disease, drug, metabolite, and toxin in their PolySearch 2 text mining tool [30]. According to the authors even though the work performs well in relation extraction, the system cannot assess the discovered relation due to lacks of training data and they are planning to improve the performance through a natural language processing (NLP) based machine learning approach. Zhou et al., proposed a knowledge-based approach Know-GENE by combining co-occurrence based gene-gene mutual information integrated with known protein-protein interactions for predicting the gene-disease associations using boosted tree regression method [31].

Recently Xu, Dong, et al., proposed a text mining tool DTMiner in which they used Stanford NER tool with dictionaries for named entity recognition and Support Vector Machine (SVM) classifier trained with local lexical and global syntax features for association detection. The authors used Genetic association databases (GAD) for evaluation and compared the results with BeFree system reported faster execution and better performance [32]. The proposed methodologies for gene disease relation extraction discussed above lacked a well-crafted supervised machine learning approach based on gold standard corpora. Among the works discussed above most of them followed a dictionary based tagging and a rule-based relation extraction. Only the two systems BeFree and DTMiner used a machine learning approach for relation extraction and reported results on EU-ADR and GAD corpora.

Due to the complexity of the gene-disease relation mentions, a limited number of the gold standard corpus and massive volume of available literature mining this relation endured as an appalling task. Machine learning based gene-disease association extraction can significantly improve the extraction and curation of genetic association of diseases. By taking all these truths on the ground, we believe that gene-disease relation extraction needs further improvement. In this work, we are proposing a methodology to this problem domain through a supervised joint ensemble learning approach using four gold standard corpora.

It has been proven that learning algorithms performed better by exploring word similarity in NLP problems using distributed representations of words [33]. Word2vec is an open source engine, which creates the distributed word representations using neural networks in the vector space by capturing both syntax and semantics characteristics [34, 35]. It explicitly encodes various patterns, which can also be represented as linear translations and linguistic regularities using two architectures namely continuous bag-of-words (CBOW) and skip gram (SG) models [33–35]. Word2vec has shown to be exhibit superior performance in multiple areas like text classification, clustering and sentiment classification [34–36].

Our proposed system solves the problem of classifying gene-disease association sentences through extensive feature engineering with word embeddings (via Word2Vec) that capture syntactic and semantic features of the domain-specific texts and jointly learned both approaches through an ensemble learning of the SVM algorithm. The feature extraction pipeline applies standard preprocessing techniques and further annotates inputs with conceptual, lexical, context related, and syntactic/semantic features. Our methodology has been tested with a composition of four standard corpora, which can act as a new baseline for future work in gene-disease relation extraction, to the best of our knowledge not been published previously.

The rest of the paper is organized as follows: Next section describes materials and methods used within this study framework followed by results and error analysis. We discussed the merits and demerits of our study in the discussion section. Finally, we concluded the paper in the last section.

## Materials and methods

In this section system architecture, data sources, feature set, detailed algorithm and methods used in this study are discussed.

### Architecture and extraction workflow

**Fig 1:**
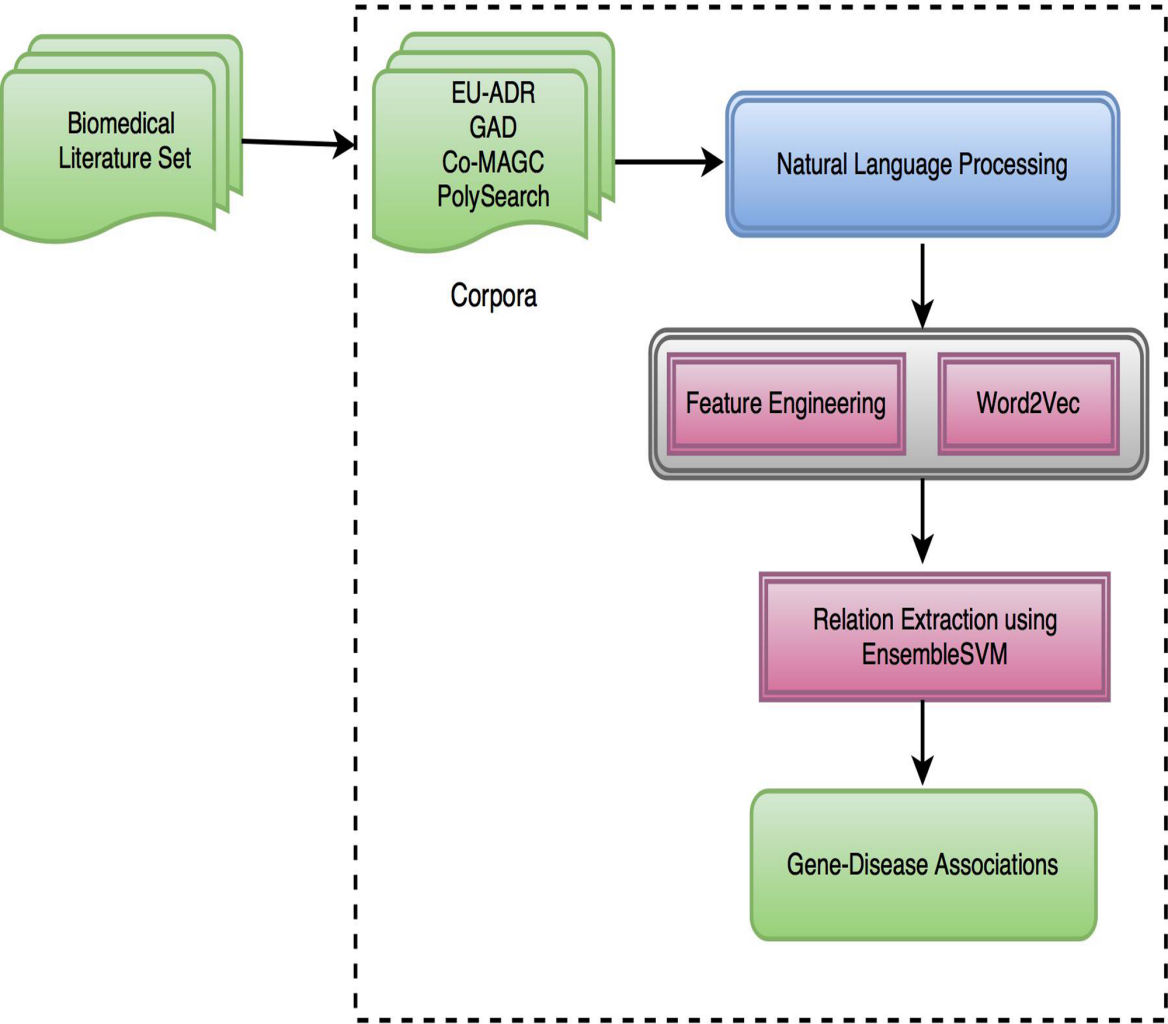
Schematic architecture of the gene-disease relation extraction system.

This study comprises of the development of a full scale supervised machine learning approach to extract gene-disease associations. We employed a joint learning strategy by combining a set of domain specific and independent syntactic and semantic features and word embedding feature Word2Vec. Subsequently, we used ensemble learning for relation extraction using SVM. Fig 1 illustrates the schematic architecture of our proposed methodology. We performed NLP followed by the generation of feature-based and Word2Vec based models. Finally, we jointly learned the models using EnsembleSVM for the extraction of gene disease relations. Further, the performance of the developed methodology was evaluated using four gold standard corpora namely EUADR, GAD, CoMAGC and PolySearch related to gene-disease relation extraction task.

**Fig 2:**
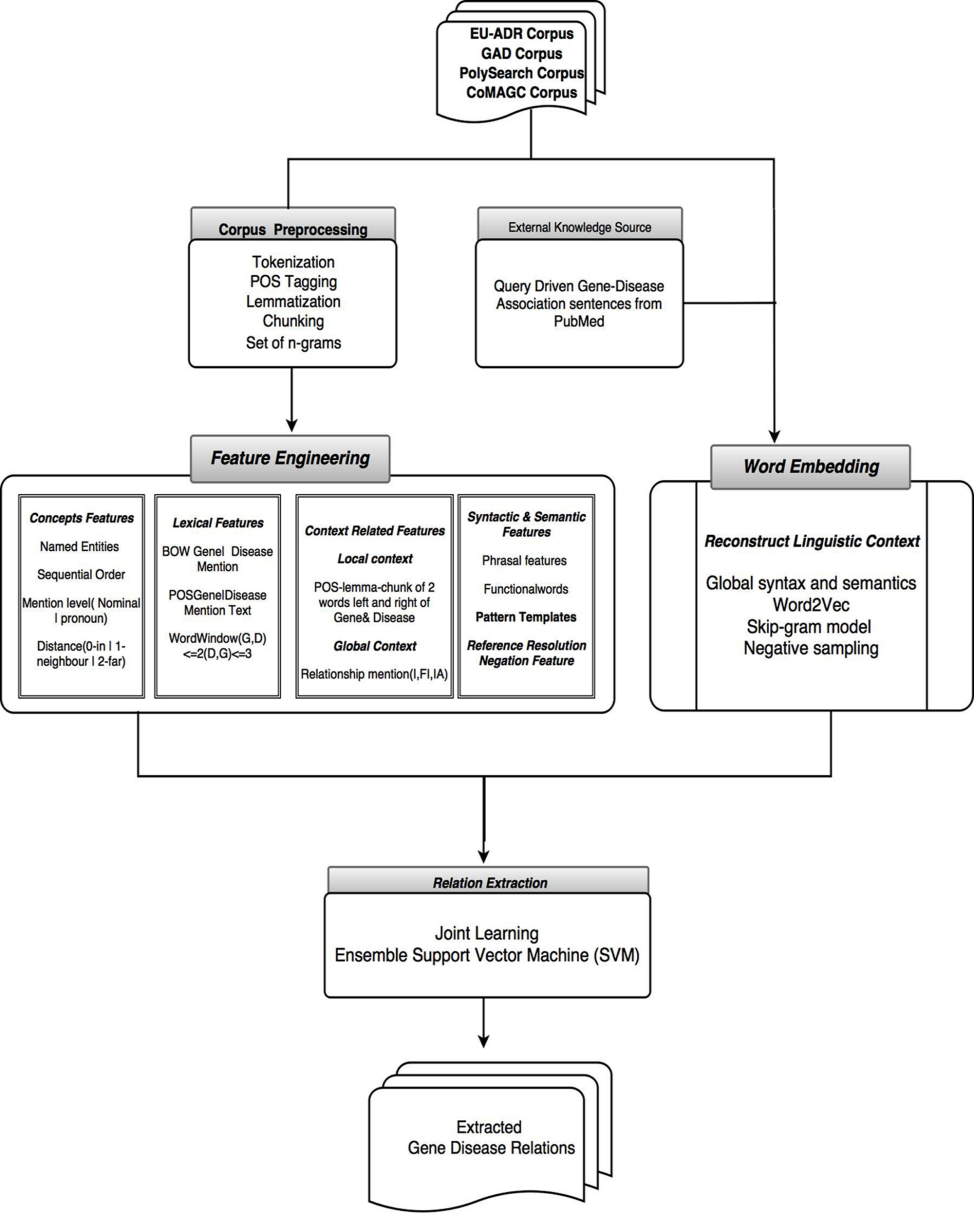
Extraction workflow of the supervised machine learning approach.

The overall workflow of the systems with details of various NLP and machine learning methods used is illustrated in Fig 2. The corpus sentences are subject to preprocessing using NLP techniques. A rich feature set is generated covering conceptual, syntax and semantic, context, lexical, pattern and negation types, used during the construction of a feature-based model. A word embedding based model is generated using Word2Vec by feeding a query-driven gene-disease associated sentences from PubMed. The Word2Vec model is created for capturing the global syntax and semantic features using the SG based model with negative sampling approach. In the final step, both the models were jointly learned using SVMs as an ensemble classifier for gene-disease relation extraction.

### Data sources

#### Gold standard corpora

To develop our supervised machine learning approach for gene-disease relation extraction, we used four corpora EU-ADR [37], GAD [38], CoMAGC [39] and PolySearch [40] for the performance evaluation of our model. We chose these corpora because all these four corpora are open source and previously studied and reported by other researchers. For example, in the BeFree [26] system, the authors used EU-ADR and GAD corpora. Similarly, in the PKDE4J [28] system, the authors reported the results on GAD and CoMAGC corpora. However, to the best of our knowledge, all four corpora not reported all together in a single study. Hence, we decided to evaluate our model for all four corpora which will form the basis for future gene-disease relation systems. Among these gold standard corpora, EU-ADR is a multi-relation annotated corpus and CoMAGC is specifically annotated for cancer. Compared to EU-ADR, GAD is a larger corpus with a high number of positive/negative gene disease associations comprises of complex disease and disorders. PolySearch corpus for gene disease associations focuses on ten specific diseases and its association to a set of 243 genes. Full Characteristics of the corpora with disease and target (Gene/Protein) has been given in Table 1. Among the corpora, three of them except CoMAGC have separately annotated positive and negative relations, and a detailed description is shown in Table 2.

**Table 1:** Corpus characteristics of full set corpora.

Corpus Characteristics	EU-ADR	GAD	CoMAGC	PolySearch
No. of Abstracts	100	5330	408	374
Total Disease Mentions	964	5330	821	522
Total Target Mentions	1664	5330	821	522
Unique Diseases	126	923	3	10
Unique Targets	213	1652	538	243
Total no. of Relations	941	5330	821	522

**Table 2:** No. of positive and negative sentences annotated in each corpus.

Corpus	EU-ADR	GAD	PolySearch
No. of Positive relations	262	2801	341
No. of Negative relations	93	2529	181

### Gene/Disease recognition

Since the corpora, GAD and PolySearch does not label all the gene, and disease mentions within each sentence, inorder to find all the mentions of both entities in every sentence, we used state of the art methodologies for it. For gene name identification, we used BANNER [41] one of the widely exploited open source implementations along with a dictionary matching procedure. For the dictionary, matching procedure, we created a gene library by integrating various sources like HGNC [42] NCBI gene database [43] and UniProtKB [20]. Since we are targeting gene/protein names and as gene and protein names are interchangeably mentioned in literature, we used both HGNC for gene names and UniProtKB for protein names.

We have already developed aNER system to tag disease names which integrates a stacked ensemble of Conditional Random Field (CRF) with the fuzzy matching of a disease dictionary [44]. In order to recognize disease names in this study, we used our above mentioned in-house developed disease name recognition system.

### Association detection

#### Feature engineering

**Fig 3:**
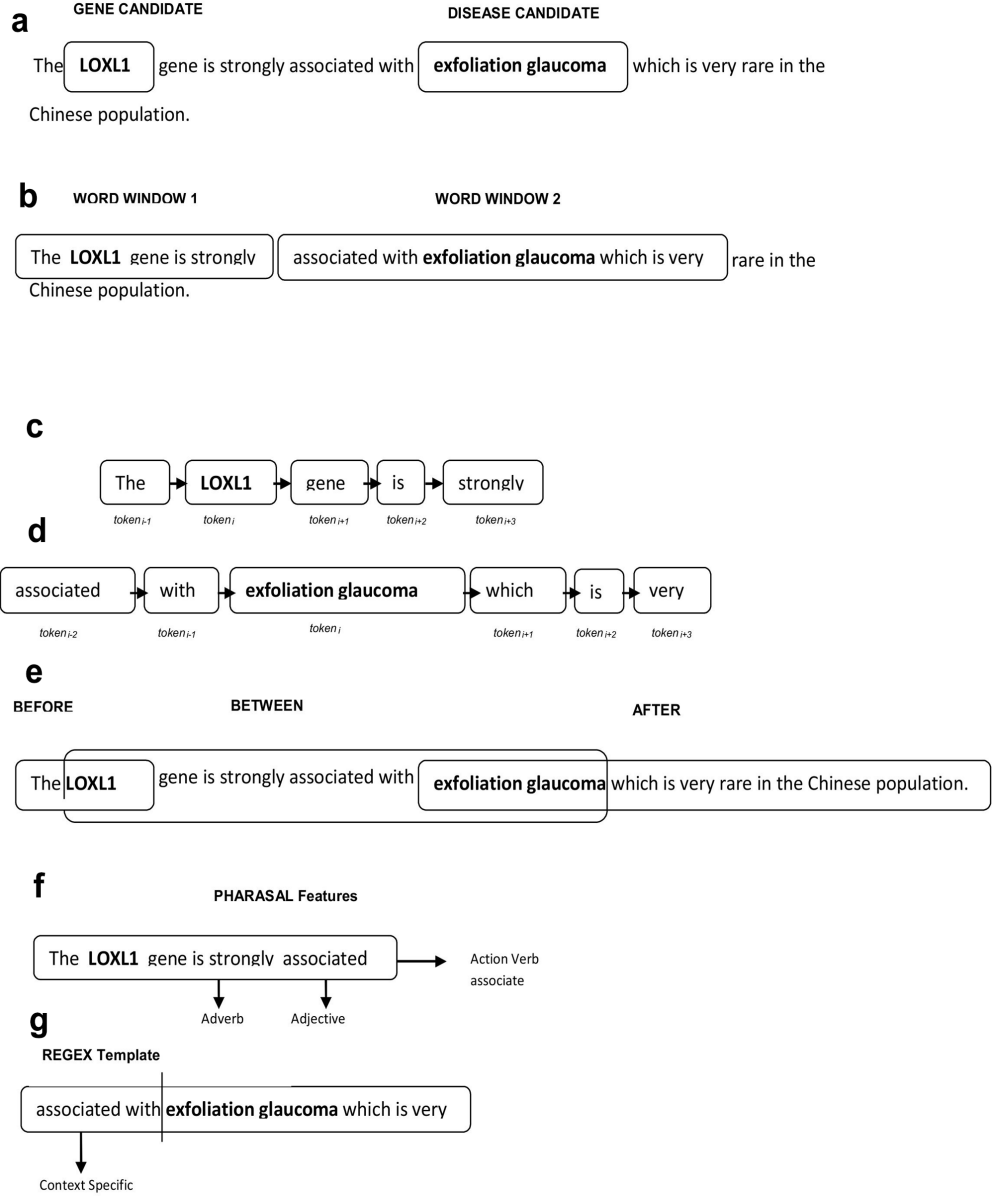
Feature representation of gene-disease relation extraction. **a)** The sentence is tagged with both LOXL1 gene and Exfoliation glaucoma disease from EU-ADR corpus with PMCID: PMC2605423 **b)** Word window representation of syntax and semantic features **c)**Tokens positioned at the left and right (n-gram) of the candidates(LOXL1 and exfoliation glaucoma)**d)**Locating the words between the entities for relational and trigger words **e)** Phrasal feature from the relational word **f)** Finding context specific word using trigger word templates.

One of the widely accepted and proven facts in machine learning, especially supervised learning is that an effective, significantly uncorrelated set of features can maximize the performance of the learning models to a large extent. In this study, our classifier uses a set of features that covers syntax and semantics of gene disease relation in both local and global level along with a set of pattern templates. A detailed description of the feature engineering applied in this work has been discussed in Table 3 followed by a feature representation in Fig 3.

**Table 3:** Full feature set used in gene-disease relation extraction.

Feature Type	Description
Syntactic & Semantic Features	Phrasal features (Verb | Noun as interaction words) Relational Keywords words Stop word removal Word Window (Useful clues about roles) (E1,A)< = 2 (A,E2)< = 3
Lexical Features	BOW Gene Mention BOW Disease Mention POS Disease Mention Text POS Gene Mention Text
Concepts Features	Named Entities (Gene & Disease names) Semantic Based Sequential Order (Gene- Disease | Disease–Gene) Gene-Disease Pair occurrence Distance (0-in | 1-neighbour | 2-far)
Context Related Features	*Local context* POS-lemma-chunk of k words left and right of Gene & Disease(k = 2) *Global Context* Relationship mention (I,FI,IA) n-Grams(n = 3) Topic Sentence Corpus frequency
Pattern Templates	Keyword trigger list Action verbs (binds, docks to, associated with) Specific Genetic phenomena s (Mutation, Haplotype information, transcriptional, phosphorylation, methylation, altered expression) Context-Specific (Cause | Effect, Treat, Indicative, Has Symptoms, Associated with, Overexpressed in, location of, predispose)
Word Representation	Word2Vec (https://code.google.com/archive/p/word2vec/)
Negation Feature	Negative independence (negation window using negation list)

### Word2Vec

One of the major complex tasks in text mining is the true representation of unstructured text into corresponding vectors in order to apply machine learning algorithms. In the recent years, linguistic research has provided ample support for the assertion that the correct vector representation of the unstructured text can significantly improve the performance of the text mining systems. Recent studies within this field provided one of the finest, largely successful new concept proposed by Mikolov et al. from Google based on deep learning called Word2Vec [33–35].

The main focus of Word2Vec is to reconstruct the linguistic context for the words by positioning the corresponding word vectors which share a common context in the given text in a high dimensional space created using the input text corpus. Word2Veccan be described as a two-layer neural net that detects similarities among word mathematically, processes the text to vector and groups the vectors of a similar word in high dimensional vector space. One important point to be noted is that the distributed vector representation of Word2Vec has been shown to carry semantic meanings [33–35]. In this paper, we used Word2Vec as a word representation feature because the generated vectors are the distributed numerical representation of the word features such as in the context of individual words like the gene-disease named entities and trigger words.

In this work, we used the Word2Vec code from Google for computing the vector space of distributed representation of gene-disease association sentences. The code provides implementation of both CBOW model and the SG model. One interesting result reported by Mikolvo et al., is that by increasing the ranging of the word window resulted in quality improvement [33–35]. By considering this, we used all the four corpora along with a query-drivendatasetof gene-disease associations from PubMed as an input to the Word2Vec tool. The tool generates a vector space by learning each vector for every word in the given gene-disease sentence vocabulary using the SG neural network architecture.

**Fig 4:**
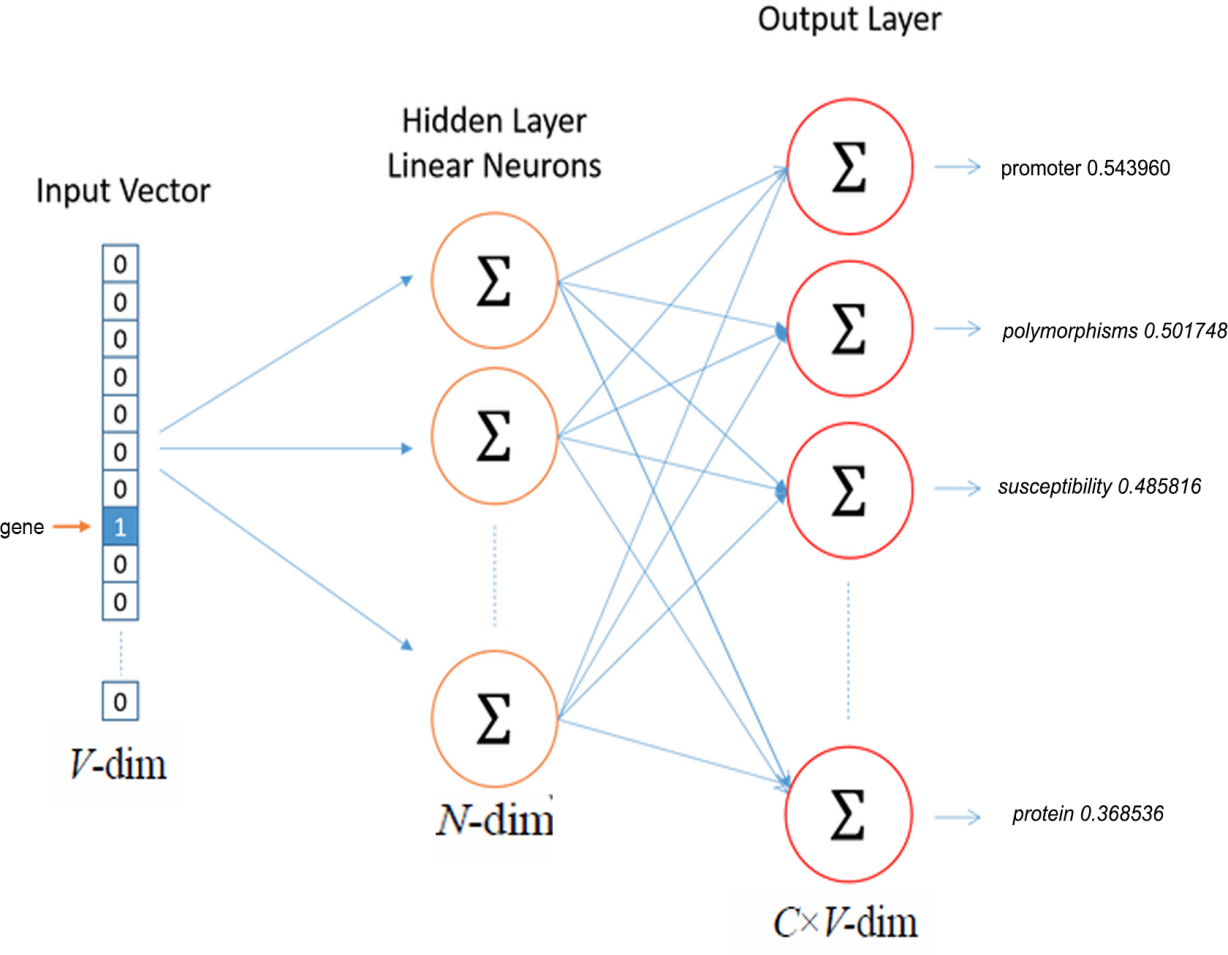
Representation of the skip-gram (SG) model with target word gene at the input layer and the learned contextual words like a promoter, susceptibility, protein etc. are in the output layer, adapted from [48].

The SG models are proposed in order to predict the current word based on the context and by utilizing another word in the same sentence improves the classification of the word [45–48]. The tool comes with a variety of tuning options like required vector dimensionality, context window size, desired training algorithm, number of threads and down sampling threshold, etc. Through literature search, we found out that the negative sampling training algorithm performs well with frequent words and low dimensional vectors. The sub-sampling parameter can improve the accuracy of the representation by training using the value 1e-5. We used the SG model with negative sampling and kept all other hyper-parameters such as word window size as 8, negative samples of 25, sub-sampling to 1e-5,20 threads and the learning rate to its default settings. The model is created at a rate of, Alpha: 0.000005, Progress: 100.11% and Words/thread/sec: 105. 31k by using 25 million (254, 224, 532) words and created a vocabulary size of 757,430 words. Upon evaluating the vector space of the model, we came to know that our model positions words like gene, promoter, polymorphisms, susceptibility and region close to each other at cosine distance of 0.543960, 0.501748, 0.485816 and 0.478719 respectively. An interesting example of the SG model representing the word 'gene' from our study has been shown in Fig 4 given below.

In the given Fig 4, above V-dim is the vocabulary size of the input layer in which one word will be there in case of skip-gram, N-dim is the size of the hidden layer and W_V x N_ is the weight between input and the hidden layer matrix and W^**'**^_N xV_is the weight between hidden and the output layer matrix. Finally, y_cj_ is the j^th^ word on the c^th^ panel of the output layer in which c = 1, 2, … …, C.

In general, the skip-gram model tries to maximize the average log probability for a given set of w_1_, w_2_, …w_N_ words as training text as given in Eq 1 below.

$$\frac{1}{N}\sum_{t=1}^{N}\sum_{-c\leq j\leq c,j!=0}\log\;p\;(w_{t+j}|w_{t})$$ (1)

In Eq 1 log probability (log p) is maximizing the context word w_t_ using the training context size c which is a function of center word w_t_ and N which is the total size of the given set of word w ranging from t = 1 to N.

### Joint learning

There has been a wider acceptance for the fact that in relation extraction keywords in the sentence can reflect the relation pattern, and the complex relations can be distinguished by the semantic properties of the given entities. In this study, we are exploring the above observation through a joint learning concept [49]. By following the joint learning method, we successfully reduced the number of domain-specific handcrafted features and correlated features. By carefully analyzing the sentences of gene disease associations, we find out that the keywords and words between the entities can reflect semantic properties of most of the relationship patterns. We also reached a conclusion that the keywords and word window representation finds very hard to distinguish the complex relation mentions, which were, in fact, can occur in case of gene disease association. In order to obtain the syntax and semantic information at a global level, we employed the concept of Word2Vec. It has been empirically reported that Word2Vec captures semantic information and in this work, we used it for global semantic relation generation.

Capturing of the local syntax and semantics of the gene-disease associations has been given in the following example. The word window patterns tagged for sentences are available in S1 File. In the given example, RELW represents the relation word GW, GWR and DW, DWR represent the gene and disease word windows in both directions.

#### Example 1

Sentence: We conclude that the <GENE> angiotensinogen M235T </GENE> gene polymorphism may be an independent predictor of <DISEASE> restenosis </DISEASE> after PTCA.

**Local syntax and semantics**: <GW-1>the</GW-1><GW-2>that</GW-2><GW-3>conclude</GW-3><GWR-1>gene</GWR-1><RELW> polymorphism </RELW><DW-1>of</DW-1><DW-2>predictor</DW-2><DW-3>independent</DW-3><DW-4>an</DW-4><DWR-1></DWR-1><DWR-2>after</DWR-2><DWR-3>PTCA</DWR-3>

For global syntax and semantics, we used ourWord2Vec word embedding model. As discussed in Word2Vec section it performs linguistic context reconstruction by positioning words of related context each other according to their cosine similarity value. The cosine similarity value is the distance measured based upon the similarity of two words. Snippet of Word2Vec output with cosine distance for word ***gene*** is given in example 2 as Table 4 below. The important cue words in the generated vocabulary with their cosine distance are provided in S2 File.

**Table 4:** The cosine similarity values generated by Word2Vec for the word ‘*gene*’.

Word	Cosine distance
Promoter	0.543960
Polymorphisms	0.501748
Susceptibility	0.485816
Region	0.478719
Receptor	0.454030
Functional	0.449036
Locus	0.433228

#### Example 2

So, we created a composite model capturing the local and global syntactic and semantic information of gene-disease associations along the contextual information through relation keywords by joint learning both feature based and word embedding based models through ensemble approach.

### Ensemble learning

SVMs are considered as one of the widely used and extensively exploited supervised machine learning algorithm with high performances reported results in classification methods [50]. SVM is based on the concept of decision planes, the one that separates set of objects with different class members that defines decision boundaries [51]. The SVM algorithm draws optimal hyperplane for linearly separable patterns and for non-linearity it uses kernel functions to transform the original data to a new dimensional space [52]. For the optimal performance, SVMs maximizes the margin of the hyperplane separation by using support vectors, the data points that lie closest to the decision surface [53]. In the present study as a binary classification problem, we used SVM to produce a supervised classification model using an optimized feature set and data with positive and negative labels as training and predicted the target values using a 10-fold cross-validation method.

Given labeled pairs of training set as (X_i,_ Y_i_) where i = 1, 2,… …n where X_i_ € R^n^ and

Y € {+1, -1}^n^, the SVM [52] can be define as an optimization problem as:

$$\min_{w,b,\xi }=\frac{1}{2}w^{T}w+C\sum_{i=1}^{n}\xi_{i}$$ (2)

Subject to

$$Y_{i}(w^{T}\varphi (X_{i})+b)\geq 1-\xi_{i}$$ (3)

$$\xi_{i}\geq 0$$ (4)

In the given Eq 3 above *X*_*i*_ represents the training vectors and *Y*_*i*_ represents the positive and negative class labels which were mapped into a higher-dimensional feature space by using the function. In Eq 2, parameter C is the classification penalty, w is the vector of coefficients and i = 1 to n represents the number of training instances. In order to handle the non-separable input data *ξ*_*i*_ is used and parameter b is a constant. In order to implement SVM we used the open-source package EnsembleSVM [54] which provides efficient routines for binary SVM ensemble models.

#### EnsembleSVM

EnsembleSVM [54] aggregates many SVM models which are trained on small subsamples of training set by employing a divide-and-conquer strategy. By engaging such a strategy EnsembleSVM successfully trains multiple base models with significantly reduced training time, which enables it in dealing with large data sets and nonlinear kernels with reduced complexity. Due to its lightweight, faster prediction and ensemble nature, this framework has been applied in a diversity of applications such as extraction of protein-protein interactions from literature, optimized audio segmentation and detection of protein complexes from protein-protein interaction networks [6,55,56]. Another motivation behind choosing EnsembleSVM is that it reduced the complexity of training procedure drastically with high prediction accuracy. In the training procedure,subsamples of the training set are bootstrapped in a bagging procedure approach and the models were aggregated through majority voting. The flexibility of the base models was maximized with instance weighted support vectors.

In this present study, we used EnsembleSVM to stratified bootstrap sampling, train multiple SVM base models on the corpora, create an ensemble of those models through aggregation and predict the accuracy using a10-fold cross-validation scenario. In the case of SVM, the feature vector makes the linear separation of data, and the kernel function is used to perform the similarity calculations faster and easier even if the feature vector is of higher dimension. In the training phase, we used RBF (Radial Basis Function) kernel as a transformation function to map our input data to a higher-dimensional space.

## Results

To evaluate the performance of the current study, we conducted a series of experiments for relation extraction on EUADR, GAD, CoMAGC and PolySearch corpora. In order to compare the performance of our proposed methodology, we compared the results with other text mining techniques, including BeFree [26], PKDE4J [28] and PolySearch2 [30].

### Evaluation metrics

We used the state-of-the-art performance measures Precision(*P*), Recall(*R*) and F-score (*F*) to evaluate the performance of our gene-disease relation extraction system. Technically type I errors are given by precision and type II errors are given by recall and F-score is the harmonic mean of precision and recall.

In general, we can define Precision (P), Recall (R), and F-score (F) as follows:

$$P=\frac{TP}{TP+FP}$$ (5)

$$R=\frac{TP}{TP+FN}$$ (6)

$$F=\frac{2*P*R}{P+R}$$ (7)

Where *TP*,FP, and *FN* are the numbers of true positives, false positives and false negatives respectively.

There has been a well-accepted practice in text mining studies is that if the gold standard corpus doesn’t come with separate training and testing set, a cross-validation scheme will be employed for reporting the results. Since all our corpora are of this type, we followed the same 10-fold cross-validation procedure employed by the previous state of the art methodologies for gene-disease relation extraction using these corpora [26,28]. We took the performance evaluations of the comparison systems as reported from literature.

### Evaluation of relation extraction

#### Corpora

We evaluated the performance of our proposed methodology on a total of four corpora in which we followed a 10-fold cross-validation strategy. Full details of the four corpora have been discussed in materials and method section, and here we are briefing the significance of the selection. Among the four corpora, EU-ADR has been annotated for multiple concepts such as gene/protein, drug, disease, and their interrelationship. As a part of the research work of BeFree system, the developers released the GAD corpus which is focusing only on gene disease relation extraction with a large number of positive negative and false associations. CoMAGC is a corpus which is specifically targeting genes associated with cancer and the causality between them. It is developed in a multi-faceted relation annotation by only focusing prostate, breast and ovarian cancers. The final corpus PolySearch is released as a part of text mining methodology PolySearch 2, a system for identifying the relationship between more than ten biological concepts. In this current study, we used these four corpora for evaluating the performance of our methodology.

#### Performance evaluation of gene disease relation extraction

We implemented a binary SVM classifier in order to automatically extract gene disease association mentioned sentences from the text. In this step, we used the tagged gene disease mentioned text along with our joint feature learning approach in order to generate the binary classifier. In general, our classifier decides a sentence S = w_1_, w_2_, …., g, …. wi, …, d, … w_n_ as a gene-disease association mention between gene (g) and disease (d). In order to train the classifier, we represented the data in the vector format with positive associations as +1 and false associations as -1.

Our classifier mainly utilizes the word embedding approach, which has been implemented through the joint learning method along with the feature set. There are some recent notable works that can be advanced to support the claim that the local global syntax and semantics features can precisely improve the performance of the relation extraction classifier [49].

As discussed earlier, word embedding has the capability to preserve semantic relation between the learned words in the vocabulary [33–35]. From this study,we got overwhelming evidence corroborating the notion that distributed word representation using Word2Vec can capture both syntactic and semantic meaning in the high dimensional vector space. Our gene-disease relation based vector representation model learned by Word2Vec successfully captured deep semantic relationship between words, especially words like *gene*, *cancer*, *mutation*, *role*, *contribution*, and *susceptibility*, etc. As discussed earlier, Word2Vec successfully reconstructed the linguistic context and when searched for word like 'tuberculosis' in our model words like *leprosy*, *mycobacterial*, *avium—intracellular*and *IFNGR1*was returned with close cosine similarity. Since all these words have a higher chance of probability to be mentioned within a sentence and especially *IFNGR1* (cosine distance-0.468307) is a gene and various study have been conducted to reveal the association between its polymorphisms and risk of tuberculosis. Another interesting result we got is related to Alzheimer's Disease (AD). The words that are in close proximity to AD in our model are *amyloid-beta*, *Alpha-2-macroglobulin*, *BIN1*, *PSEN1*, *CYP46*, *neuroinflammation*, *rs3818361*, *rs2986017*, and *K-variant*. Among these, most of them are genes or some form of genetic polymorphisms that are extensively studied for its key role in AD. The model deeply captured both syntactic and semantic associations among gene-disease related words and can play a significant role in predicting the future possible association. By analyzing these results, it is evident that this word embedding approach played a significant role by in our gene-disease relation extraction methodology for achieving superior performance.

By carefully examining the gold stand corpora, we also came to a conclusion that the trigger words and negative association words can act as a major player in this classification task.

#### Example 3

These results strongly suggest that the g.-247C/T polymorphism in the <GENE>**CHI3L1**</GENE> promoter region is associated with the risk of <DISEASE>**atopy**</DISEASE>

In the above example 3 the trigger word associated exactly three-word window from gene *CHI3L1*and disease *atopy*. The words connecting gene to disease are promoter region and risk, which were syntactically and semantically depends upon the word associated.

#### Example 4

These results suggest that the C1772T polymorphism in <GENE>**HIF-1alpha**</GENE> is not involved in progressionor metastasis of<DISEASE>**colorectal carcinoma**</DISEASE>

In the above example 4, the word ‘not’ completely reversed the context of the associative sentence from a gene-disease relation mention to a false association. We found out that the word ‘not’ in ‘not associated’ or ‘not involved’ or the word ‘hard’ with ‘hardly any evidence’ or the word ‘no’ with ‘no confirmation’ or ‘no evidence’ strongly indicated a false gene disease association. In order to capture this information, we used trigger word and negation word lists to tag these words. We strongly believe that by giving more feature weights to these important words and representing them as positive and negative values enable us to build a robust classifier.

As described earlier, the joint learning approach combines syntax and semantic features at local and global level by including trigger words, n-grams and word windows as given in example1 as feature-based model along with word embedding model. After all the features were extracted, we used EnsembleSVM [54] for building the binary classifier. EnsembleSVM performs an aggregation on basic SVM models and builds a final single ensemble classifier. The RBF kernel functions in SVM treat the entire given feature vectors as a bag of words. All the given labels are associated with appropriate weights and an indication of positive or negative values and finally, an ensemble of base models is created.

**Fig 5:**
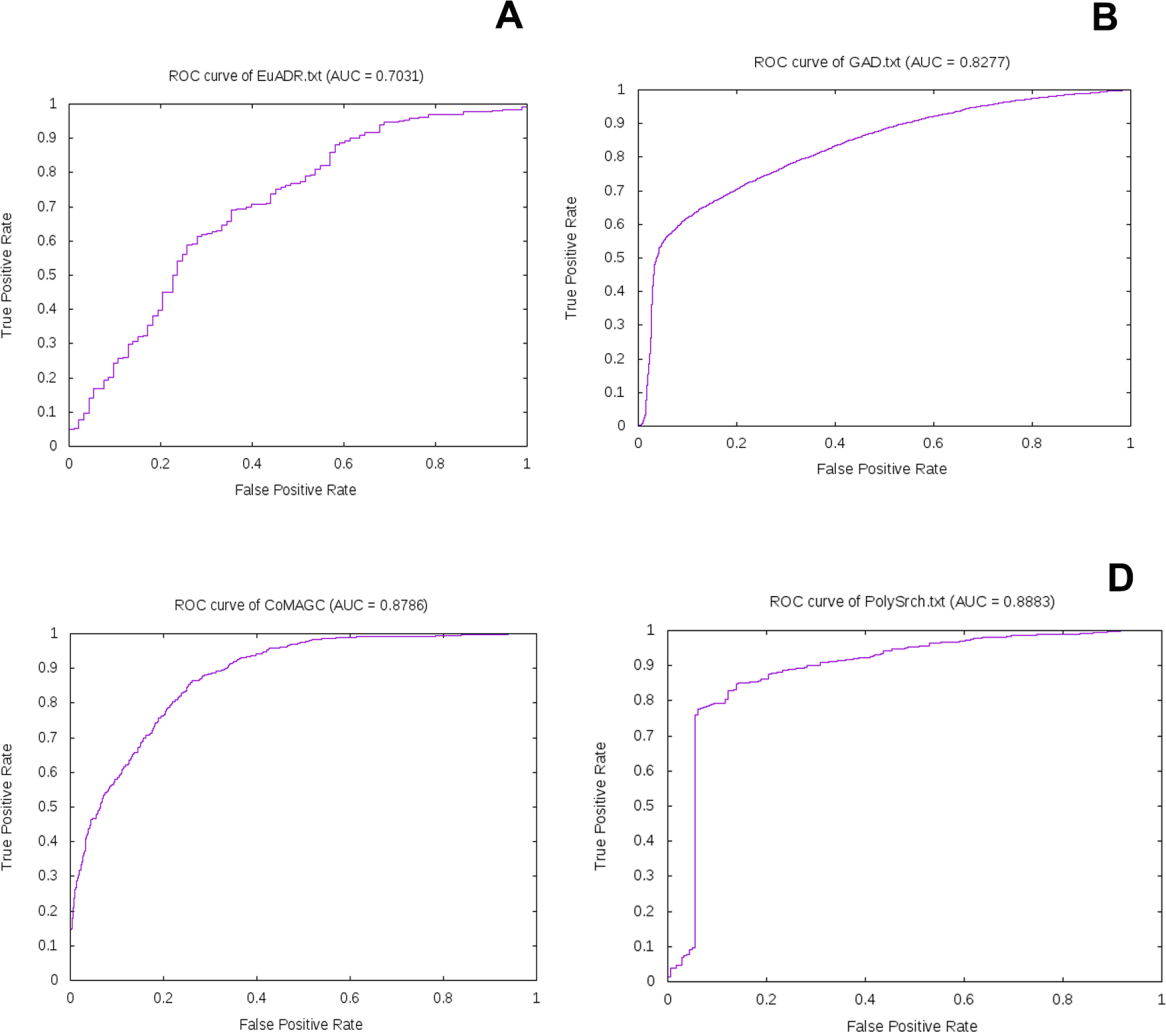
ROC with respect to FPR and TPR on four corpora upon 10-fold cross-validation. In this figure, a, b, c, and d represents the receiver operating curves of EU-ADR, GAD, CoMAGC and PolySearch corpora respectively.

For performance comparison of our methodology, we used the reported results of BeFree [26], PKDE4J [28] and PolySearch2 [30] text mining methodologies. We have done a 10-fold cross validation in all the corpora and as a baseline result, we got comparative precision values. Upon doing an ensemble learning with all the feature weights our model achieved 85.34%, 83.93%, 87.39% and 85.57% of F-measure on EUADR, GAD, CoMAGC and PolySearch corpora. A detailed representation of precision, recall, and f-score for all the corpora have been given in Tables 5 to 7. For better representation results, we plotted ROC (Receiver Operating Curve) curves with respect to false positive rate (FPR) and true positive rate (TPR) and gave below in Fig 5.

**Table 5:** Performance comparison of the proposed system with the BeFree [26] system.

Corpus	System	Precision(%)	Recall(%)	F-Score(%)
**EU-ADR**	**Proposed System**	**76.43**	**98.01**	**85.34**
	BeFree [26]	75.10	97.70	84.6
**GAD**	**Proposed System**	**79.21**	**89.25**	**83.93**
	BeFree [26]	77.80	87.20	82.20

**Table 6:** Performance comparison of the proposed system with the PKDE4J [28] system.

Corpus	System	Precision(%)	Recall(%)	F-Score(%)
**CoMAGC**	**Proposed System**	**81.89**	**93.70**	**87.39**
	PKDE4J [28]	71.5	88.00	78.80
**GAD**	**Proposed System**	**79.21**	**89.25**	**83.93**
	PKDE4J [28]	-	-	83.80

**Table 7:** Performance comparison of the proposed system with the PolySearch2 [30] system.

Corpus	System	Precision(%)	Recall(%)	F-Score(%)
**PolySearch**	**Proposed System**	**83.45**	**87.82**	**85.57**
	PolySearch2[30]	87.08	90.91	88.95

**Fig 6:**
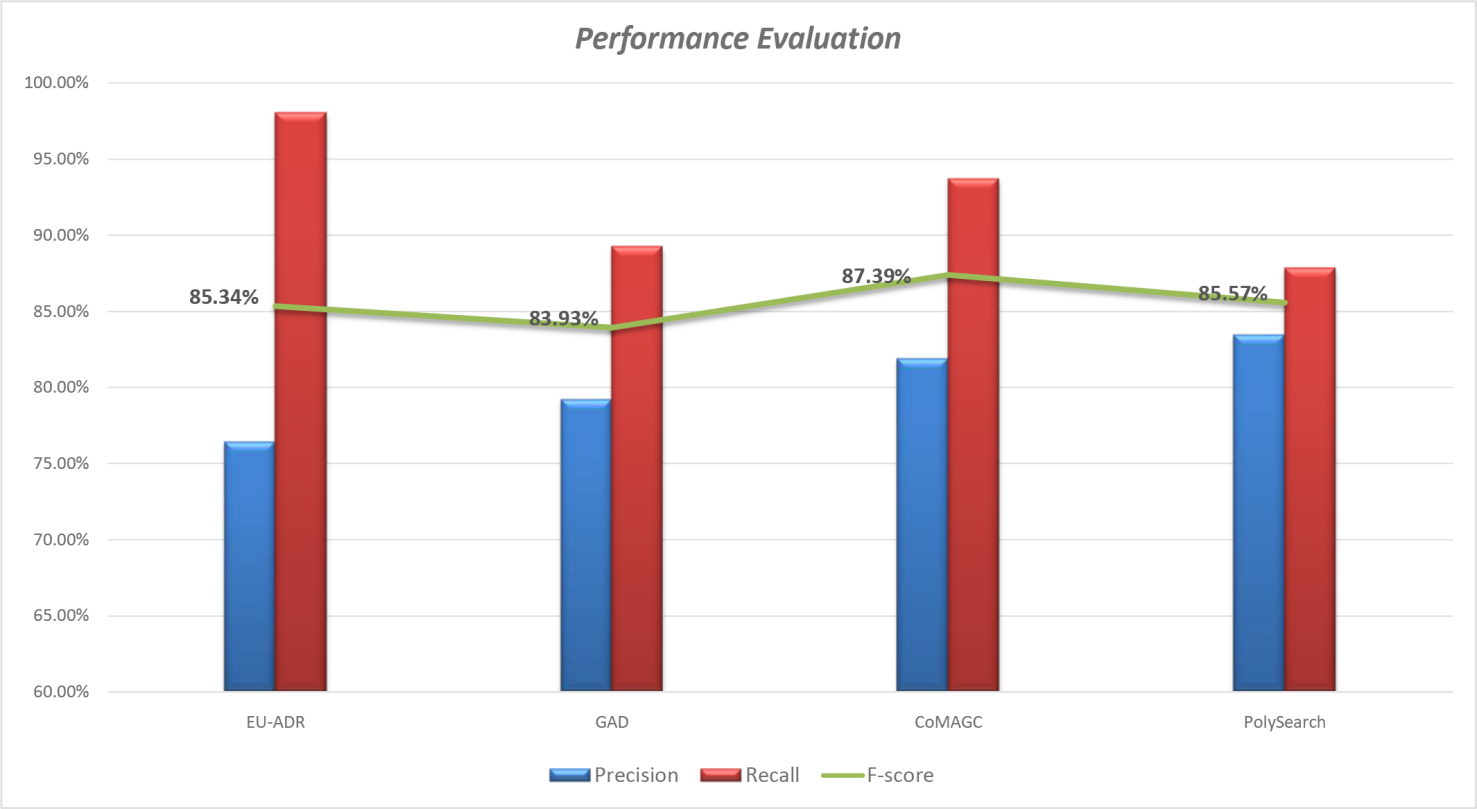
Performance evaluation of gene disease relation extraction on four different corpora.

**Fig 7:**
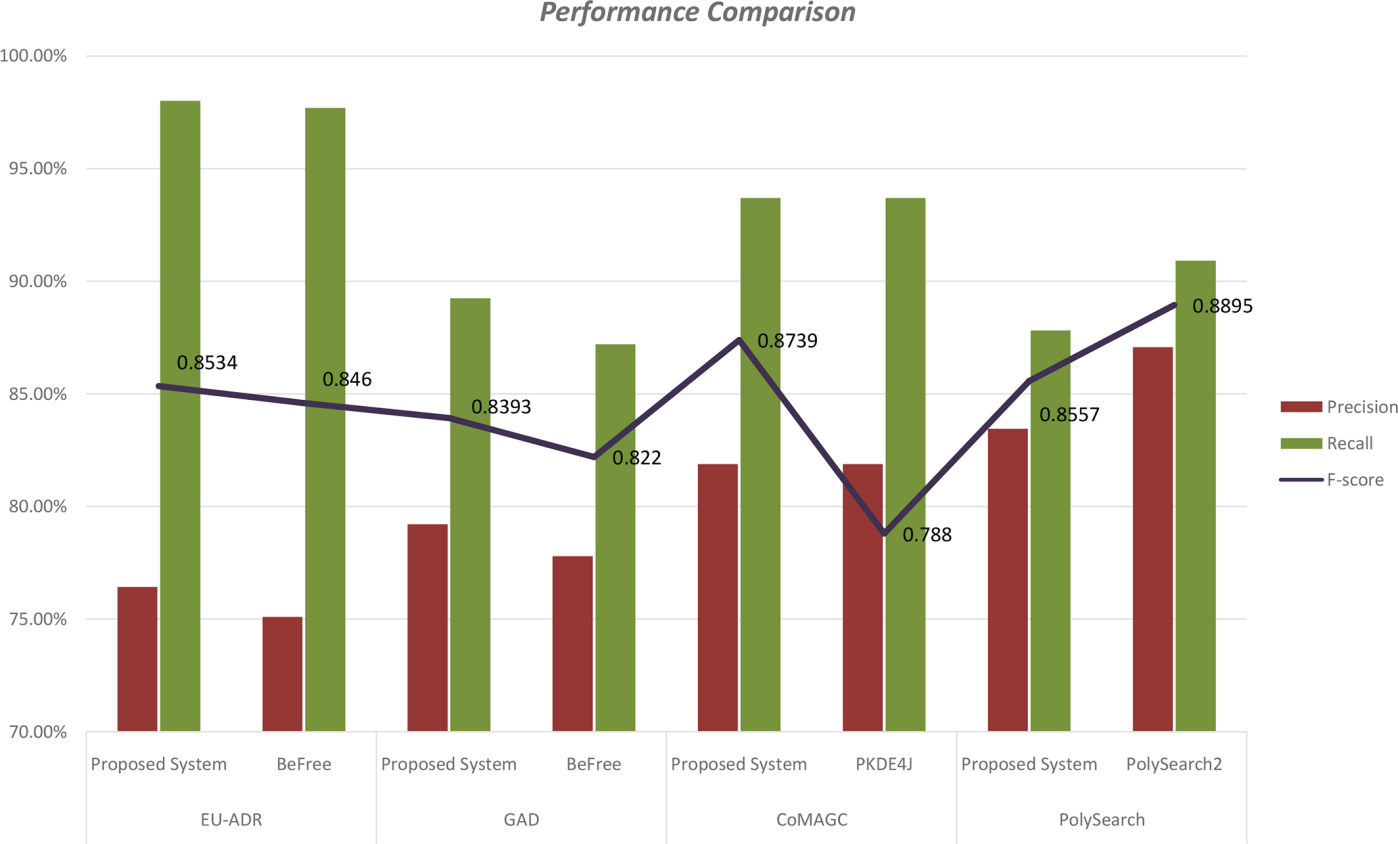
Performance comparison of gene disease relation extraction on four different corpora.

In general, our methodology exhibits comparative performance with F-score values ranging from 81 to 89%. We used BeFree systems to compare the result on EU-ADR corpus, and we got a slightly improved F-score due to the improvement in precision. Our methodology achieved 84% and 89% F-scores in an experiment with GAD, and CoMAGC compared to PKDE4Js 83% and 78%of F-scores. In both the cases, we can see a high precision, and low recall value increases. Although no reported machine learning approaches are there in PolySearch corpus, we compared our results with PolySearch2 dictionary matching methodology. Even though a direct comparison is not necessary, our methodology achieves 3% less F-score on PolySearch corpus, and we achieved a comparative recall, and this shows a promising result. A detailed graphical representation of performance evaluation and comparison for our classifier has been given in Figs 6 and 7.

### Error analysis

There seems to be no compelling reason to argue that even with comparative performance our system also produces minimal errors. Even though our approach of word embedding method gave superior performance it comes with some drawbacks. The major one is that Word2Vec learned prepositions and articles such as *'of'*, *'between'* and *'the'* to the vocabulary but skipped medical terms like *'HCNPpp'* and *'IL23R'*. Since Word2Vec comes with no rules, it is not possible to regulate this issue. We tried removing the stop words, but it drastically reduced the performance because preposition plays an important role in giving syntactic and semantic meaning to the sentence. Another issue we faced is the words were learned along with the commas or semicolon like 'cancer,' or 'cancer;' adding it as a new word thereby reduced the possibility of another useful medical term to be added to the vocabulary.

To figure out the errors generated by the proposed methodology, we manually examined the prediction file and considered the false positive and false negative sentences mention gene-disease relation. Upon evaluating we found out that many negative results occurred due to the complexity of gene disease relation mentions with long sentences.

#### Example 5

Although rs7566605 was not significantly associated with obesityin our study population, we cannot rule out the involvement of <GENE>INSIG2</GENE> in <DISEASE>obesity</DISEASE> related traits as we found a significant association of another tag SNP in <GENE>INSIG2</GENE> with both BMI and ABDCIR.

In the above sentence in order to explain the relationship between gene *INSIG2* and disease *obesity*, the authors mentioned a single conjunction and used multiple references with connective words a coma. Our classifier identified this as a negative relation resulting in a false positive.

#### Example 6

Our findings suggest that the <GENE>p53</GENE> codon 72 polymorphism is unlikely to be associated with <DISEASE>endometriosis</DISEASE> in Japanese women

Since our classifier is focusing on syntax and semantics, a negative word ‘unlikely’ made the above sentence a false relation whereas our classifier unable to detect it. It is also worth mentioning that our classifier detected some other negative words like ‘rarely’, ‘hardly’ and ‘no evidence’.

#### Example 7

In summary, our results show that <DISEASE> cystinuria </DISEASE> is a complex disease which is not only caused by mutations in <GENE> SLC7A9 </GENE> and SLC3A1 but also influenced by other modifying factors such as variants in SLC7A9

In the above-givenexample, the gene disease association has been mentioned using a “not only… but also” conjunction and the sentence of this type of co-relative type has been detected as a negative sentence by our classifier.

## Discussion

We have developed a supervised machine learning approach for extracting gene disease association mention sentences from literature. The specialties of the proposed methodology are that to the best of our knowledge, we are the first one to report gene disease relation extraction results on four corpora otherwise have been reported separately in multiple studies. Secondly, we also integrated a shallow word embedding approach Word2Vec, which has empirically proven to hold semantic meanings. We strongly believe that by exploiting syntactic and semantic properties in bothlocal and global context made our methodology to achieve competitive performance. Although the reconstruction of the linguistic context approach of word embedding captures both syntax and semantics information, in case of the words which are not in the vocabulary (unknown entities) Word2Vec performs nothing. In this scenario, our feature engineering module covering lexical, concepts, context related syntactic and semantic features along with pattern templates captured these entities thereby solving the unknown word entity problem from word embedding. As discussed earlier in the Word2Vec and result section, words describing the same relation come with lesser cosine distance as a result close to each other, on the contrary, word pairs describing non-related sentences come with maximum cosine distance and placed far from each other.

Upon evaluating the performance of our methodology, we achieved competitive results in all four corpora for a machine learning based approach. Our system achieved a 0.74 to 8.59% improvement on F-score with an average of 85.55% with the almost high recall in all corpora. The main reason behind the systems elevated recall is the integration of word embedding approach with the dictionary matching of contextual keywords and negation words. It is also worthy to mention that even though we are using fewer but representative features our word embedding Word2Vec approach can give an added advantage to our system. Since it is focusing on reconstructing the linguistic context along with semantic meaning, all the related mentions of concepts like gene, disease, associations, etc. were positioned closely. It enables the classifier to easily understand the context of unknown sentences with gene disease association mentions with any synonyms. Furthermore, we also came to a conclusion that our NER methods able to give a high performance in the gene and disease name recognition which in turn made the association detection task a less complex.

A disadvantage of our proposed methodology is that it does not perform well for long complex sentences. As discussed in the error analysis section if gene and disease names are mentioned in multiple times with single connectives or in the case of not only but also sentences our system unable to detect the relation. In practice for such an association mentioned sentence, we could introduce some method that can dissect and reduces the complexity of the sentence. Possible future enhancements are mainly focusing on reducing the complexity of the sentence and making the classifier to deal better with negative sentences.

## Conclusions

The effectiveness of genome-wide associations studies (GWAS), genome-wide expression studies(GWES), successfulness of guilt-by-association (GBA) approach and the fast-evolving of sequence technologies are some of the compelling reasons behind a large number of research publications mainly focusing on genomic variations of diseases. In order to discover knowledge from this vast amount of literature, we propose a supervised machine learning system which automatically extracts gene disease relations from it. In this study, for gene disease relation extraction task we designated an effective set of features, which covers both local, global syntax and semantics of gene disease association and built a robust SVM binary classifier. Further, the performance of the system was first time evaluated with four gold standard corpora available in gene-disease association extraction task. Our system with an effective feature set and a robust SVM classifier achieved proportionate performance, reported good balance in accuracy and exhibited improved F-score in comparison with existing state of the art systems, which were evaluated on few corpora only. Overall, our competitively performed methodology and evaluation with four data sets will form a baseline for future gene associated disease tasks.

## Supporting information

S1 File Local syntax and semantics from the gene-disease association sentences. (TXT)

S2 File Word projections of Word2Vec model generated using cosine similarity as closeness. (DOCX)
