# Supplementary material for: Automatic extraction of gene-disease associations from literature using joint ensemble learning
Source: PLoS One. 2018 Jul 26;13(7):e0200699. doi: 10.1371/journal.pone.0200699 (PMC6061985; doi:10.1371/journal.pone.0200699)
Supplement: S2 File — (DOCX) [file pone.0200699.s002.docx]

**Word projections of Word2Vec model generated using cosine similarity as closeness in this study are listed below. For each word a set of words are learned by the model based on deep syntactic and semantic analysis.**

##############################################################################

For the word ‘***gene***’

| Word | Cosine distance |
| --- | --- |
| Promoter | 0.543960 |
| polymorphisms | 0.501748 |
| susceptibility | 0.485816 |
| Region | 0.478719 |
| Receptor | 0.454030 |
| functional | 0.449036 |
| Locus | 0.433228 |

For the word ‘***disease***’

| Word | Cosine distance |
| --- | --- |
| Diseases | 0.628460 |
| Disease | 0.483733 |
| Condition | 0.453703 |
| illness | 0.403629 |
| Disorders | 0.399636 |
| degeneration | 0.384478 |
| Rheumatic | 0.349869 |
| Parkinson's | 0.349117 |

For the word ‘***association***’

| Word | Cosine distance |
| --- | --- |
| relationship | 0.674816 |
| correlation | 0.571134 |
| Between | 0.503780 |
| Relation | 0.493939 |
| contribution | 0.475045 |
| interaction | 0.452992 |
| connection | 0.448478 |
| Link | 0.437904 |

For the word ‘***cancer***’

| Word | Cosine distance |
| --- | --- |
| Cancers | 0.729805 |
| carcinoma | 0.688322 |
| adenocarcinoma | 0.612205 |
| carcinogenesis | 0.525469 |
| cancer-related | 0.542381 |
| Ovarian | 0.484365 |
| Breast | 0.480661 |
| Gastric | 0.477836 |
| esophageal | 0.468960 |
| pancreatic | 0.460512 |
| colorectal | 0.445053 |

For the word ‘***role***’

| Word | Cosine distance |
| --- | --- |
| roles | 0.814547 |
| implication | 0.692367 |
| involvement | 0.666551 |
| contribution | 0.630750 |
| importance | 0.594812 |
| pathogenesis | 0.568588 |
| link | 0.562416 |
| relevance | 0.553413 |
| connection  influence | 0.530692  0.520449 |
|  |  |

For the word ‘***susceptibility***’

| Word | Cosine distance |
| --- | --- |
| predisposition | 0.740993 |
| predisposing | 0.603899 |
| Risk | 0.557073 |
| genetic | 0.515563 |
| pathogenesis | 0.506489 |
| development | 0.506043 |
| etiology | 0.488728 |
| conferring  variations  low-penetrance  non-HLA | 0.455816  0.441551  0.426161  0.417734 |

For the word ‘***Alzheimer's***’

| Word | Cosine distance |  |
| --- | --- | --- |
| Alzheimer | 0.926816 |  |
| Parkinson's | 0.676775 |  |
| Creutzfeldt-Jakob | 0.567305 |  |
| AD | 0.555302 |  |
| Huntington's | 0.523755 |  |
| (PS-1) | 0.523053 |  |
| (BIN1) | 0.514735 |  |
| (CLU)  K-variant  Integrator  non-HLA | 0.512926  0.494948  0.426161  0.486328 |  |
| macroglobulin | 0.479502 |  |
| (ACT) | 0.478853 |  |
| Neuroinflammation | 0.475970 |  |
| Amyloid-β | 0.463162 |  |
| alpha2-macroglobulin | 0.463137 |  |
| butyrylcholinesterase | 0.460772 |  |
| neurodegenerative | 0.453702 |  |
| neprilysin | 0.450970 |  |
| Q7R | 0.450821 |  |
| rs3818361 | 0.448411 |  |
| PSEN1 | 0.448116 |  |
| rs2986017 | 0.445491 |  |
| CYP46 | 0.443935 |  |
|  |  |  |

For the word ‘***cause***’

| Word | Cosine distance |
| --- | --- |
| causes | 0.823047 |
| causing | 0.685723 |
| responsible | 0.590065 |
| caused | 0.557545 |
| form | 0.548463 |
| leading | 0.496942 |
| occur | 0.493529 |
| contributor | 0.491833 |
| induce | 0.453250 |
| affecting | 0.443814 |
| causative | 0.440416 |
| lead | 0.420475 |
|  |  |

For the word ‘***marker***’

| Word | Cosine distance |
| --- | --- |
| markers | 0.700900 |
| indicator | 0.643301 |
| biomarker | 0.591697 |
| predictor | 0.463260 |
| adjunct | 0.426152 |
| surrogate | 0.425893 |
| bio-marker | 0.414934 |
| factor | 0.394520 |
| locus | 0.386867 |
| marker-assisted | 0.376612 |
| D6S273 | 0.369470 |
| prognosticator | 0.362022 |
| D17S579 | 0.340281 |
| 8p22 | 0.339284 |
| endophenotype | 0.338555 |
| S100P | 0.330275 |

For the word ‘***allele***’

| Word | Cosine distance |
| --- | --- |
| genotype | 0.664578 |
| Alleles | 0.643616 |
| haplotype | 0.568260 |
| T-allele | 0.529806 |
| A-allele | 0.525729 |
| variant | 0.523417 |
| C-allele | 0.517218 |
| homozygous | 0.516802 |
| genotypes | 0.513323 |
| TC+CC | 0.511769 |
| rs1063857 | 0.495729 |
|  |  |

For the word ‘***disorder***’

| Word | Cosine distance |
| --- | --- |
| disorders | 0.721717 |
| condition | 0.617781 |
| illness | 0.577505 |
| symptoms | 0.514028 |
| neuroimmunological | 0.502327 |
| psychotic | 0.478039 |
| symptomatology | 0.465791 |
| disturbances | 0.464454 |
| Schizophrenia | 0.459905 |
| genodermatosis | 0.451876 |
| chronic | 0.436368 |
|  |  |

For the word ‘***genetic***’

| Word | Cosine distance |
| --- | --- |
| polygenic | 0.628691 |
| environmental | 0.554103 |
| phenotypic | 0.523535 |
| etiological | 0.517918 |
| susceptibility | 0.515563 |
| Multifactorial | 0.514005 |
| immunogenetic | 0.498568 |
| heritable | 0.497689 |
| inter-individual | 0.457887 |
| epigenetic | 0.450868 |
| inherited | 0.449709 |
| pharmacogenetic | 0.449191 |
| epidemiological | 0.448886 |
| molecular | 0.448832 |
| genetical | 0.447080 |
| Pathophysiological | 0.430787 |
| aetiological | 0.427557 |
|  |  |

For the word ‘***SNP***’

| Word | Cosine distance |
| --- | --- |
| SNPs | 0.721626 |
| haplotype | 0.597349 |
| variant | 0.514532 |
| tagSNP | 0.511545 |
| tSNP | 0.506263 |
| intronic | 0.478118 |
| rs16969968 | 0.461779 |
| rs40401 | 0.457620 |
| imputed | 0.455703 |
| rs10757278 | 0.453136 |
| SEC8L1 | 0.448056 |
| RND3 | 0.438074 |
| SNPS | 0.435121 |
| locus | 0.430500 |
| rs1333049 | 0.427407 |
| Single-marker | 0.421809 |
|  |  |

For the word ‘***factor***’

| Word | Cosine distance |
| --- | --- |
| determinant | 0.576769 |
| indicator | 0.487485 |
| variant | 0.514532 |
| tagSNP | 0.511545 |
| tSNP | 0.506263 |
| intronic | 0.478118 |
| rs16969968 | 0.461779 |
| rs40401 | 0.457620 |
| imputed | 0.455703 |
| rs10757278 | 0.453136 |
| SEC8L1 | 0.448056 |
| RND3 | 0.438074 |
| SNPS | 0.435121 |
| locus | 0.430500 |
| rs1333049 | 0.427407 |
| Single-marker | 0.421809 |
|  |  |

For the word ‘***overexpressed***’

| Word | Cosine distance |
| --- | --- |
| over-expressed | 0.746519 |
| upregulated | 0.656656 |
| overexpression | 0.583718 |
| up-regulated | 0.560981 |
| anti-apoptosis | 0.470329 |
| high-grade | 0.463678 |
| ERα-positive | 0.454579 |
| hPRL | 0.445776 |
| immunostaining | 0.440313 |
| OCT4 | 0.439624 |
| HER4 | 0.435321 |
| MYBL2 | 0.434675 |
| ADAM15 | 0.432892 |
| DEK | 0.432621 |
| miR-34 | 0.430081 |
|  |  |

For the word ‘***tuberculosis***’

| Word | Cosine distance |
| --- | --- |
| TB | 0.801078 |
| leprae | 0.650321 |
| PTB | 0.631534 |
| Tuberculosis | 0.608321 |
| avium | 0.585592 |
| avium-intracellulare | 0.566908 |
| leprosy | 0.559593 |
| Dectin-2 | 0.554865 |
| mycobacterial | 0.530649 |
| Zahedan | 0.520804 |
| ulcerans | 0.492864 |
| brucellosis | 0.491876 |
| cryptococcosis | 0.487446 |
| malaria | 0.485603 |
| leishmaniasis | 0.427407 |
| pMAH135 | 0.481323 |
| MAC | 0.480507 |
| MRC1 | 0.473363 |
| MDR-TB | 0.469255 |
| CISH | 0.469153 |
| IFNGR1 | 0.468307 |
| bovis | 0.463593 |
| 1513A/C | 0.463093 |
| leptospirosis | 0.459261 |
|  |  |
